# Tuning Fullerene Intercalation in a Poly (thiophene) derivative by Controlling the Polymer Degree of Self-Organisation

**DOI:** 10.1038/srep34609

**Published:** 2016-10-04

**Authors:** G. M. Paternò, M. W. A. Skoda, Robert Dalgliesh, F. Cacialli, V. García Sakai

**Affiliations:** 1 London Centre for Nanotechnology, Department of Physics and Astronomy, University College London, Gower Street, London WC1E 6BT, UK; 2 ISIS Pulsed Neutron and Muon Source, Science and Technology Facilities Council, Rutherford Appleton Laboratory, Harwell Science and Innovation Campus, Didcot OX11 0QX, UK

## Abstract

Controlling the nanoscale arrangement in polymer-fullerene organic solar cells is of paramount importance to boost the performance of such promising class of photovoltaic diodes. In this work, we use a pseudo-bilayer system made of poly(2,5-bis(3-hexadecylthiophen-2-yl)thieno[3,2-b]thiophene (PBTTT) and [6,6]-phenyl-C_61_-butyric acid methyl ester (PCBM), to acquire a more complete understanding of the diffusion and intercalation of the fullerene-derivative within the polymer layer. By exploiting morphological and structural characterisation techniques, we observe that if we increase the film solidification time the polymer develops a higher crystalline order, and, as a result, it does not allow fullerene molecules to intercalate between the polymer side-chains. Gaining insight into the detailed fullerene intercalation mechanism is important for the development of organic photovoltaic diodes (PVDs).

One of the most important advantages of organic semiconductors over their inorganic counterparts lies in the possibility of tailoring their physical and chemical properties by exploiting the power of intermolecular interactions. However, to take full advantage of this and hence optimise the performance of related opto-electronics devices, such as organic photovoltaic diodes (PVDs), thin film transistors (TFTs) and light-emitting diodes (LEDs), it is important to gain a detailed understanding on the way these carbon-based materials interact and arrange at the nanoscale^1^,^2^. For instance, exciton diffusion and splitting, charge transport and recombination in polymer-fullerene bulk heterojunction (BHJ) solar cells are strongly dependent on the nano- and meso-scale structure^3^ and dynamics^4^ of the interpenetrated network formed by the two semiconductors.

More specifically, in recent years it has been proved that polymer: fullerene solid blends are complex systems, in which both pure polymer and fullerene crystalline phases^5^,^6^ and amorphous intermixed phases coexist^7^. Interestingly, McGehee and collaborators have shown that fullerene derivatives can intercalate between the side-chains of a variety of polymers when there is enough space between the polymer side-chains^8^ and the fullerene derivative is sufficiently small to fit between them^9^. In the case of semicrystalline polymers, it has been noted that fullerene can intercalate into the polymer crystalline phase forming stable bimolecular polymer-fullerene crystals^10^,^11^,^12^. The formation of such a mixed and well-ordered phase plays a central role in determining the optimum polymer: fullerene ratio in BHJ solar cells for efficient excitons splitting and charges generation, as also it has been found by molecular simulations^13^. Due to fullerene intercalation prevailing over phase separation in these systems, a pure electron-transporting phase is only formed when the fullerene loading exceeds the quantity needed for full intercalation.

In this letter, we show that the degree of intercalation of the fullerene derivative [6,6]-phenyl-C_61_-butyric acid methyl ester (PCBM) in the semicrystalline polymer poly(2,5-bis(3-hexadecylthiophen-2-yl)thieno[3,2-b]thiophene (PBTTT) depends significantly on the degree of self-organisation of the polymer, which in turn is controlled by the film solidification rate during processing. The temperature-dependent solubility of PBTTT^14^,^15^, insoluble at room temperature but highly soluble in hot chlorinated solvents (above 70 °C), allowed us to make bilayers of these components by using a sequential-processing techniques^16^,^17^,^18^. Thus, we were able to prepare first the polymer layer from a hot solution, and then we overlaid a fullerene layer from the same solvent at room temperature, without dissolving the underlying polymer layer. We cast the polymer via three different deposition techniques to explore different solvent evaporation rates: spin-coating, slow-drying and drop-casting. The resulting films exhibited different morphologies and structural features, with PBTTT drop-cast exhibiting the highest crystalline order. By employing morphological and structural characterisation techniques such as atomic force microscopy (AFM), x-ray diffraction (XRD) and by investigating the out-of-plane segregation of the two components by means of neutron reflectivity (NR), we observe that the rate of fullerene intercalation and formation of bimolecular crystals can be decreased substantially by increasing the crystallinity of the pre-deposited PBTTT films. This eventually leads to large differences in terms of electrical features, as revealed by the characterisation of photovoltaic diodes incorporating these different films as active material.

## Results and Discussion

**Figure 1:**
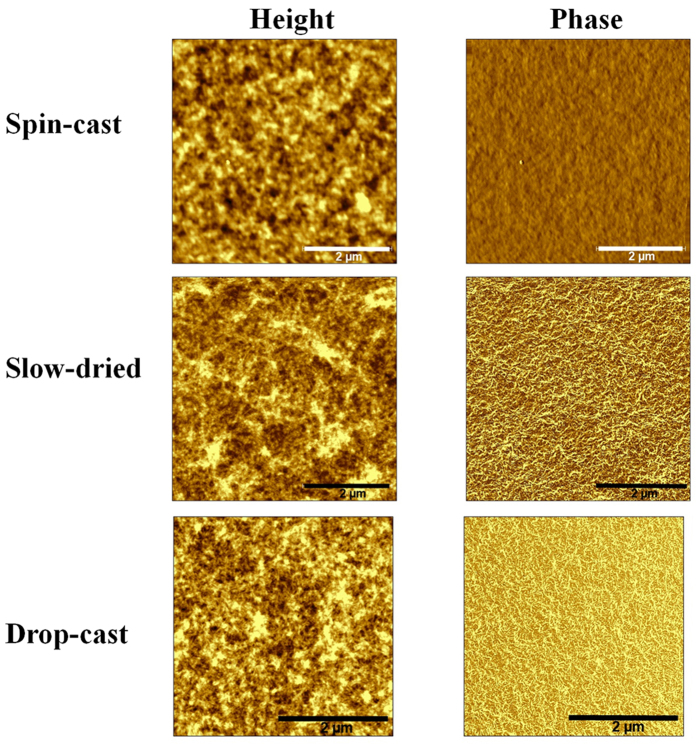
AFM height and phase images of pure PBTTT spin cast, slow-dried and drop-cast height. The height and phase scales are: 0–15 nm and 0–15 deg. for PBTTT spin-cast, 0–30 nm and 0–20 deg. for slow-dried and 0–30 nm and 0–20 deg. for drop-cast. The film thickness is approximately 60 ± 10 nm for all the pure polymer samples (measured by profilometer). None of the pure PBTTT films were thermally annealed.

Figure 1a shows the AFM height images alongside their phase pictures for pure PBTTT cast via spin-casting, slow-drying and drop-casting depositions. We can observe clear morphological differences among the three films, which suggest that the polymer degree of self-organisation can be varied by controlling the film solidification time. In particular, the solvent evaporation time was evaluated by visual inspection of the change of the film colour (light to dark purple) when it changes from the liquid to the solid phase. For spin-cast films, such change in colour occurred in ~10 seconds, although we spun it for 60 seconds to dry the film further. In the case of the “slow-drying” deposition, we spun-cast the film for 5 seconds and then placed the film, while still wet, in a closed Petri dish to complete the solidification process, which occurred in ~2 minutes. For drop-cast films, we placed a droplet of the PBTTT solution onto the substrate and waited ~12 hrs until the film solidified. The deposition procedure was optimised to achieve the same thickness for all three samples, 60 nm ± 10 nm. We can observe that, whereas the spin-cast film looks relatively unstructured, the slow-dried one show fibril-like aggregates that are more distinctively visible in the phase images. The height and relative amount of such nanofibrillar features increased further when the film was deposited via drop-casting. Such an increased ordering when increasing the film solidification time is provided indirectly by the root mean square (RMS) roughness of the films^19^, which increases in the order spin-cast → slow-dried → drop-cast (2.42, 5.20 and 5.59 nm, respectively).

**Figure 2:**
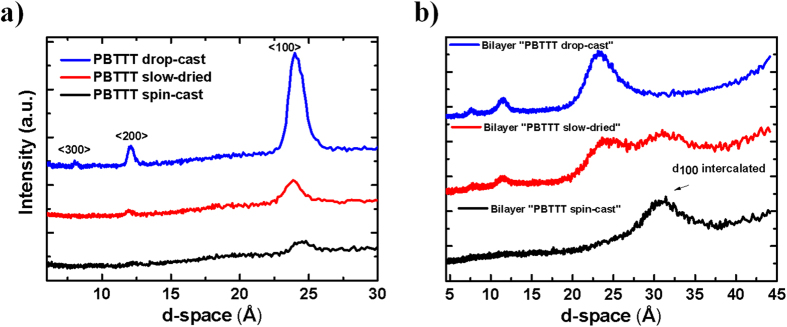
(**a**) XRD pattern (out-of-plane) of PBTTT pure deposited via spin-casting, slow-drying and drop-casting. The <100> peak (at ~24° for all the films) and its higher orders refer to the lamellar stacking (see ref. 12). None of the pure PBTTT films was thermally annealed. (**b**) XRD pattern (out-of-plane) of PBTTT/PCBM films obtained by sequentially depositing the PCBM on top of the three different polymer films (PBTTT spin-cast, slow-dried and drop cast). Although perfect bilayers are not formed due to PCBM diffusion and intercalation in the polymer layer, we refers to these samples as bilayer “PBTTT spin-cast”, “PBTTT slow-dried” and “PBTTT drop-cast” for simplicity. Note that all the bilayers were annealed at 185 °C for 10 minutes.

The x-ray diffraction pattern of the pure PBTTT films cast via spin-casting, slow-drying and drop-casting (Fig. 2a) indicates clearly that the degree of order increases with the film solidification time, as indicated by the increase of the peak intensity and decrease of the FWHM of the <100> lamellar peak, following the order spin-casting → slow-drying → drop-casting.

Now we turn to the characterisation of the PBTTT - PCBM bilayers, obtained by depositing the PCBM layer on top of the three different PBTTT films (spin-cast, slow-dried and drop-cast). All the bilayers were thermally annealed at the polymer glass transition (185 °C) for 10 minutes. We refer to these samples as “PBTTT spin-cast”, “PBTTT-slow-dried and “PBTTT drop-cast” bilayers. The addition of PCBM causes a clear expansion of the PBTTT lamellar peak because of fullerene intercalation^10^, and thus we can use XRD as a tool to determine the degree of PCBM intercalation and correlate this with the PBTTT morphology. The diffraction pattern in Fig. 2b shows that, whereas in the pure polymer the spacing for the lamellar peak is 24.0 ± 0.2 Å, in the “PBTTT spin-cast” the d-spacing has increased to 30.9 ± 0.1 Å, indicating that full PCBM intercalation occurs for this sample and bimolecular crystals are formed. On the other hand, for the “PBTTT drop-cast” bilayer, the plot shows the same pattern as for the pure polymer. Interestingly, for “PBTTT slow-dried” bilayer both the pure polymer peak and bimolecular crystalline peak coexist, suggesting that PCBM partially intercalates in between the polymer side-chains and full intercalation is not achieved. It is interesting to note that a similar trend in the XRD pattern can be observed when the degree of intercalation is controlled by varying the fullerene loading in PBTTT: PC_71_BM blends, with full intercalation occurring already with a 1:1 ratio, partial intercalation with a 4:1 ratio and no intercalation with lower PCBM loadings^10^. Although this corroborates our findings, it should be mentioned that in our case fullerene intercalation is intrinsically controlled by the degree of self-organisation of the polymer, and does not depend on the fullerene loading. In particular, it seems that when we favour the formation of a stable polymer crystalline phase, pre-PCBM loading, by employing a deposition techniques with slower solvent evaporation rates (i.e. slow-drying and drop-casting), the polymer does not allow the fullerene molecules to diffuse within the polymer layer and intercalate in between the side-chains. Therefore, by simply varying the film solidification time and hence the degree of polymer self-organisation, we can easily control the degree of fullerene intercalation.

**Figure 3:**
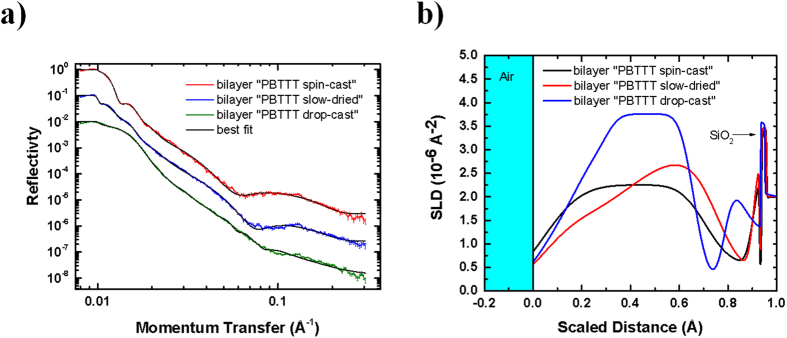
NR curves (**a**) and SLD profiles (**b**) for “PBTTT spin -cast”, “PBTTT slow -dried” and “PBTTT drop -cast” bilayers. Note that the x-axis was normalised to thickness, with a scale from 0 (air interface) to 1 (substrate interface). The total thicknesses of “PBTTT spin-cast” and “PBTTT slow-dried” bilayers are ~120 nm, and ~80 nm for “PBTTT drop-cast” bilayer. All samples were annealed at 185 °C for 10 minutes.

Having established that fullerene intercalation can be tuned by controlling the polymer’s degree of self-organisation, we now turn to specular NR investigation, which allows us to gain insight into the vertical composition of the layer. NR is an ideal tool to monitor the vertical segregation in polymer-fullerene blends^20^,^21^,^22^,^23^: the strong interaction between neutrons and hydrogenated organic materials is a clear advantage over X-rays, as is the lack of potential beam damage. In addition, the scattering length density (SLD) contrast between the PBTTT and PCBM components is large, almost an order of magnitude (5.59 × 10^−7^ Å^−2^ and 3.76 × 10^−6^ Å^−2^, respectively). In Fig. 3 the reflectivity curves and the relative SLD depth-profiles for the three different samples are presented. Note that in all the reflectivity profiles the Kiessig fringes, caused by the interference of waves reflected in multi-layered systems, are not clearly visible. In our samples those fringes are likely being damped by the high roughness between the various interfaces in the multi-layer system. To confirm that the damping of such fringes are mainly due to the high interfacial roughness, likely caused by PCBM diffusion and intercalation, we simulated the expected NR profiles with a small value of interfacial roughness (taken from AFM). In the case of low roughess the fringes appear (see Supplementary Figure S2). In addition, to rule out the possibility of poor lateral homogeneity of the sample, we performed further NR measurements on different smaller illuminated areas (10 mm), which show no appreciable differences as a function of probed region (Supplementary Figure S1). However, by taking advantage of the large difference between the SLD of PBTTT and PCBM, we were able to obtain information about the vertical composition using the calculated SLD for pure PBTTT and PCBM. In particular, the SLD profiles normal to the film were obtained by fitting the reflectivity curves using a model of slabs and were normalised to thickness. Note that the mean square roughness of the bilayers obtained via AFM (2 nm, 3 nm and 0.2 nm for bilayer “PBTTT spin-cast”, “PBTTT slow-dried” and “PBTTT drop-cast”, respectively) is sensibly lower than the ones obtained by means of NR (15 nm, 13 nm and 9 nm for bilayer “PBTTT spin-cast”, “PBTTT slow-dried” and “PBTTT drop-cast”, respectively), although the trend follows the same order. We attribute this to the larger probed length in NR than AFM (10 μm).

The SLD profiles reveal a complex vertical PBTTT-PCBM stratification that appears to be function of the different PCBM intercalation features in the bilayers. In general, we observe two PBTTT: PCBM mixing regions: a first region close to the air interface that is likely produced upon PCBM-PBTTT contact, and a deeper mixing region that is formed after further PCBM vertical diffusion towards the bulk of the layer. Note that PCBM diffusion and preferential segregation towards the substrate is favoured by its higher surface energy^23^,^24^ than poly (thiophene) derivatives. In this context, moving from the air interface (0) to the substrate interface (1) for bilayer “PBTTT spin-cast” we can observe: (i) a BHJ-like region with a PBTTT: PCBM ratio of 1:1 (~80 nm); (ii) a PBTTT enriched layer (~24 nm); two thin and relatively rough wetting layers close to the SiO_2_ interface. In particular, the first wetting layer (3 nm) with a ~1:1 PBTTT: PCBM ratio can originate from the further diffusion and mixing of the non-intercalated PCBM with PBTTT segregated at the bottom of the layer, whereas the last wetting component (1 nm) consist of a pure PBTTT layer. It is worth noting that the formation of a PBTTT wetting layer a the substrate interface would be beneficial for the operation of solar cells, since this avoids the segregation of the electron-acceptor component at the anode, where it would hamper hole extraction.

On the other hand, for the “PBTTT drop-cast” bilayer PCBM the intercalation process does not seem to take place effectively, as we can observe a PCBM enriched top surface (~40 nm) and a PBTTT-rich region (15 nm). As the amount of non-intercalated PCBM is higher in this case, the further diffusion of PCBM towards the substrate leads to rougher and more PCBM-enriched wetting layers. Interestingly, the vertical structure of the bilayer “PBTTT slow-dried” is more complex as it can be considered as an “hybrid” between the two aforementioned samples, with (i) a first PBTTT-PCBM 4:1 BHJ region (~40 nm), (ii) a PCBM-rich region (~35 nm), (iii) a PBTTT-rich region (~15 nm) and (iv) two thin wetting layers whose PBCM enrichment is slightly higher than in bilayer “PBTTT spin-cast” but lower than in bilayer “PBTTT drop-cast”. These features can be seen clearly in the simulated SLD profiles for which the interfacial layer roughness values are ten times lower than the experimental ones (see Supplementary Figure 2).

In any case, it is interesting to point out the NR results also agree with the XRD data, suggesting a strong dependence of the fullerene intercalation rate on the polymer degree of order. In addition, the calculated compositions of the top layers fit the results in the study by Mayer *et al*.^10^ as a function of PCBM loading, which shows that partial intercalation occurs with a 4:1 polymer:fullerene ratio, whereas the fullerene fully intercalates when the ratio is 1:1.

**Figure 4:**
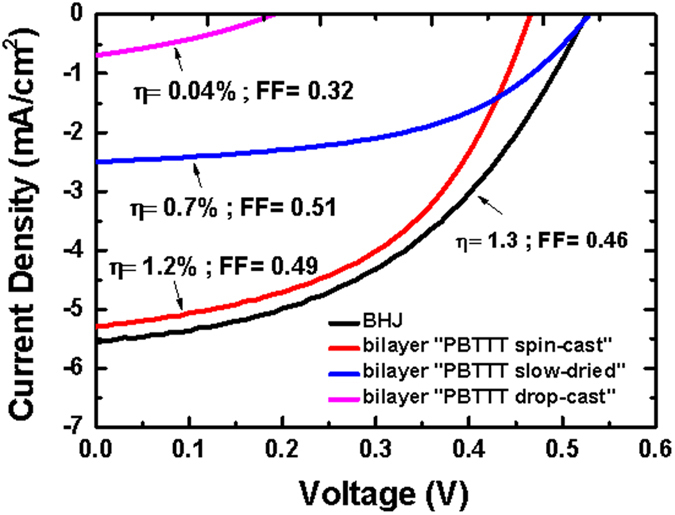
J-V characteristics for the PBTTT: PCBM pseudo bilayers. The BHJ (PBTTT/PCBM weight ratio 1:3) J-V characteristic is also presented for comparison.

We now discuss how this ordering relates to the electronic performance of the solar cells. Figure 4 shows the J-V characteristics for the three PBTTT/PCBM devices. We can observe that the performace of “PBTTT spin-cast” bilayer device approaches that of a PBTTT: PCBM BHJ with a ratio of 1:3 (efficiencies of 1.2% and 1.3%, respectively). This further confirms that if the polymer degree of self-organisation is low, PCBM can diffuse significantly inside the polymer layer and intercalate, leading to a BHJ-like structure. Interestingly, it can be also noted that, even though the polymer-fullerene ratio within the “PBTTT spin-cast” bilayer is 1:1 and hence well below the optimum ratio^25^,^26^ for PBTTT: PCBM, the efficiency is still comparable for these two types of devices. The slight losses in current and open-circuit voltage for the “PBTTT spin-cast” bilayer, which are likely due to the lower polymer-fullerene interfacial area, might in fact be counterbalanced by the better vertical segregation of the two components in such pseudo-bilayers devices, in which the electron-donor material preferentially segregates at the anode, as revealed by NR measurements. On the other hand, if the polymer is already well-structured, as in the case of PBTTT slow-dried and PBTTT drop-cast, it does not allow fullerene molecules to intercalate and, therefore, charges cannot be efficiently extracted due to the low polymer-fullerene contact area. This results in poor efficiencies, with the drop-cast sample giving the worst performance. The poorer performances of these two samples and, in particular, of the bilayer “PBTTT drop-cast” device, can be also related to the unfavourable vertical arcitecture in these samples, which show a stronger PCBM segregation at the substrate interface. It is important to note that the charge generation process in OPVs not only depends on the vertical phase separation and stratification, but also on the lateral morphology in the active layer. For instance, the fact that the “PBTTT drop-cast” bilayer has the larger crystallites sizes, larger than the typical exciton diffusion length (10 nm)^27^, leads to a smaller fraction of split excitons at the PBTTT-PCBM interface and a lower amount of free charges collected at the electrodes. This would cause a lower efficiency for this sample.

## Conclusion

In summary, we have shown that the degree of fullerene intercalation is not only a function of the fullerene size and the free volume available between the polymer side-chains, but also of the degree of self-organisation of the polymer prior to mixing with the PCBM. In particular, our findings suggest that if we assist the polymer in the development of a stable thermodynamic state by slowing down the solvent evaporation rate during the film solidification, the polymer does not permit the fullerene to intercalate and mix at the molecular scale. Although the degree of intercalation in PBTTT: PCBM scales with the solar cell performance, in not-intercalated systems the electron-donor/electron-acceptor interfacial area available for excitons splitting and charges generation is reduced significantly. Therefore, shedding light on the mechanism of intercalation can be paramount for the optimisation of polymer-fullerene solar cells and, more in general, for controlling the opto-electronic properties in a variety of organic-based devices.

## Materials and Methods

### Solar cells

C16-PBTTT (Ossila) and PC_61_BM (Aldrich) were used without further purification. PBTTT/PCBM solar cells were prepared by depositing the active layer on indium tin oxide (ITO) pre-patterned glass substrates. The ITO substrates were sonicated in acetone and isopropyl alcohol for 10 minutes, dried in a flow of dry nitrogen and then placed in an oxygen-plasma for a 10 min treatment^28^. Oxygen plasma has been shown to increase work function, as well as surface polarity and energy, and thereby help adhesion of subsequent (especially polar) layers to be deposited on top. A 30 nm thick hole-injection layer of poly (3,4-ethylenedioxthiophene): poly (styrene sulfonate)(PEDOT-PSS) was spin-coated from aqueous solution and baked at 150 °C for 10 min, to increase work function and facilitate hole-extraction^29^. The active layer was obtained by first depositing the polymer from hot ODCB 20 mg/ml solution (70 °C) either via spin-coating, slow-drying or drop-casting (see main text for details), followed by a spin-cast deposition of PCBM (20 mg/ml) from the same solvent but at room temperature. For PBTTT spin-cast films, the warm solution was deposited onto the substrate and spun for 60 seconds at 4000 rpm. For slow-dried films, PBTTT was firstly spun for 5 seconds at 4000 rpm and then put it in a Petri dish to complete the film growth. The drop-cast films were obtained by depositing a droplet of solution (0.1 mg/mL in DCB) onto the substrate and letting the solvent dry naturally. To prevent formation of a defect-rich interface due to the quick crystallization of the solution upon contacting the cooler substrate, the substrates were warmed to 70 °C before deposition. A PCBM layer was then spun on top of those films (20 mg/mL DCB, 800 rpm for 60 seconds), yielding thicknesses of 80 ± 10 nm for all the samples, as measured by profilometry. Although a perfect bilayer may not be formed due to some diffusion and intercalation of PCBM into PBTTT, we refer to these as (nominally) “bilayer samples” for brevity. Finally, the Al cathode was thermally evaporated on the active layer in a high vacuum chamber (~2.5 × 10^−6^ mbar). All devices were annealed by placing them on a hot plate at 185 °C for 10 minutes in a nitrogen glove box. The photovoltaic efficiency was measured with a Sun 3000 solar simulator (110 * 110 mm^2^ area), equipped with a Xenon lamp and an air mass (AM) 1.5 G filter (Abet technologies class AAA).

### XRD and AFM

XRD measurements were performed with a Rigaku SmartLab diffractometer (Rigaku, Tokyo, Japan) at the ISIS Neutron and Muon Facility, Rutherford Appleton Laboratory, UK, by using a K_α_ wavelength emitted by a Cu anode (0.154 nm) and a Cross Beam Optics (CBO). To avoid beam defocusing, the measurements were carried out in parallel beam mode in parallel beam mode. All XRD diffraction patterns were collected using a symmetrical out-of-plane θ/2θ configuration.

AFM images were recorded with a Veeco Dimension in tapping mode, using NSC35/AIBS ultra sharp cantilevers (MikroMasch Europe).

### Neutron Reflectivity

Neutron reflectivity measurements were carried out at the Offspec neutron reflectometer also at ISIS, using a specular scattering geometry^30^. The Offspec reflectometer used incidents wavelengths of neutrons from 1.5 to 14.5 Å and a time-of-flight detection system. Three incident angles (0.35°, 1° and 2.3°) were used to obtain the reflectivity as a function of the momentum transfer perpendicular to the sample plane, q_z_ = (4π sin(θ))/λ The scattering length densities (SLD) for PBTTT and PCBM are 5.59 × 10^−7^ Å^−2^ and 3.76 × 10^−6^ Å^−2^, respectively. For NR measurements, the polymer/fullerene layers were cast onto Si/SiO_2_ wafers with a diameter of 50 mm, following the same deposition procedure used for OPVs fabrication. However, given the larger substrate size in comparison with OPVs samples we had to lower the deposition speed to 2000 rpm for “PBTTT spin-cast” and “PBTTT slow-dried” samples. This led to slightly thicker layers for those two samples (120 ± 10 nm for both bilayer “PBTTT spin-cast” and “PBTTT slow-dried”), whereas the measured thickness of the “PBTTT drop-cast” bilayer was comparable with the solar cell one (80 ± 10 nm). We did not observe any dewetting phenomena during and after films deposition. Eventually, the samples were thermally annealed at 185 °C for 10 minutes in vacuum. The NR data were analysed using the software RasCal (version 1.1.3, A. Hughes, ISIS Spallation Neutron Source) which employs an optical matrix formalism based on the Abeles method^31^. In this approach, the interface is divided into a series of slabs, each of which is characterized by its SLD, thickness, and roughness. The interfacial roughness between adjacent layers was included in the model as an error function of standard deviation (Nevot-Croce approach)^32^. Statistical analysis showed that for the NR fits, a model with four layers was necessary to describe the data (SiO_x_ and three layers for the “bilayer” region). The interfacial roughness between these layers was significant compared to their thickness, indicating a relatively gradual change in the composition of the layer. A multibox model can be used for systems with large roughnesses and can provide a much more accurate description in terms of number of layers, parameters and compositions. However, the conclusions presented here will not be affected and such a detailed model description will constitute further work.

## Additional Information

**How to cite this article**: Paternò, G. M. *et al*. Tuning Fullerene Intercalation in a Poly (thiophene) derivative by Controlling the Polymer Degree of Self-Organisation. *Sci. Rep*. **6**, 34609; doi: 10.1038/srep34609 (2016).

## Supplementary Material

Supplementary Information
