# Supplementary material for: Tuning Fullerene Intercalation in a Poly (thiophene) derivative by Controlling the Polymer Degree of Self-Organisation
Source: Sci Rep. 2016 Oct 4;6:34609. doi: 10.1038/srep34609 (PMC5048155; doi:10.1038/srep34609)
Supplement: Supplementary Information [file srep34609-s1.pdf]

Supplementary Information for

Tuning Fullerene Intercalation in a Poly (thiophene) derivative by Controlling the Polymer Degree of Self-Organisation

G. M Paternò[1], M.W. A. Skoda[2], Robert Dalgliesh[2], F. Cacialli[1] and V. García Sakai[2]

[1]London Centre for Nanotechnology, Department of Physics and Astronomy, University College London, Gower Street, London WC1E 6BT, UK

[2]ISIS Pulsed Neutron and Muon Source, Science and Technology Facilities Council, Rutherford Appleton Laboratory, Harwell Scie nce and Innovation Campus, Didcot OX11 0QX, UK

Supplementary Figure 1

To check the lateral homogeneity of the films, we run NR measurements with a smaller illumination footprint. Here we show a comparison of the results from two different footprints for the "PBTTT spin-cast" sample. The reflectivity profiles show no appreciable differences, with the exception of a higher noise for the less illuminated sample (black curve), making 1mm the smallest slit chosen. In addition, although not shown, we also performed some additional XRR measurements on different spots in the sample (eg. middle and to the sides, with a slit opening of 5mm) and we also saw no differences, which agrees with the NR results.

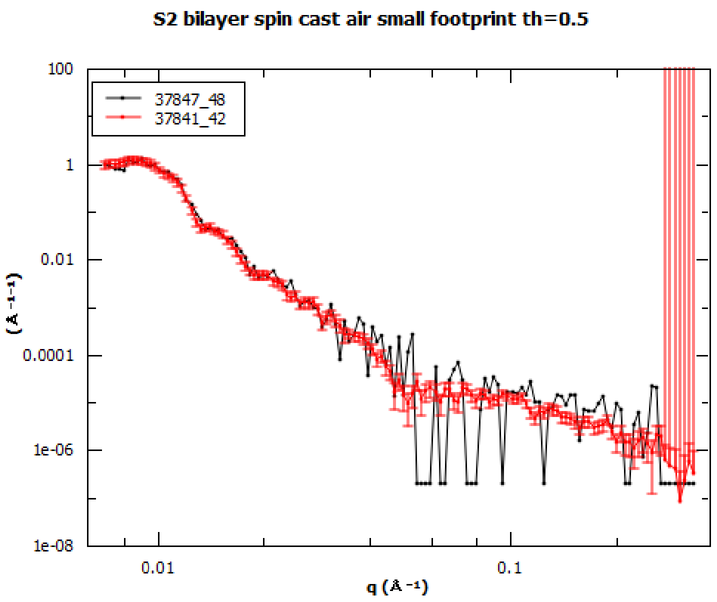

Supplementary Figure S1: NR profiles of the bilayer "PBTTT spin-cast" taken with 10 mm (black curve) and 30 mm (red curve).

Supplementary Figure 2

We simulated the NR profiles of all the samples, to confirm that the damping of Kiessig fringes are mainly due to the high interfacial roughness in such pseudo-bilayers systems. As a starting value for the roughness parameter, we chose the experimental values obtained via AFM, which are around one order of magnitude lower that the NR roughness. These simulated curves allow us to appreciate better the different vertical stratification of the two components throughout the films.

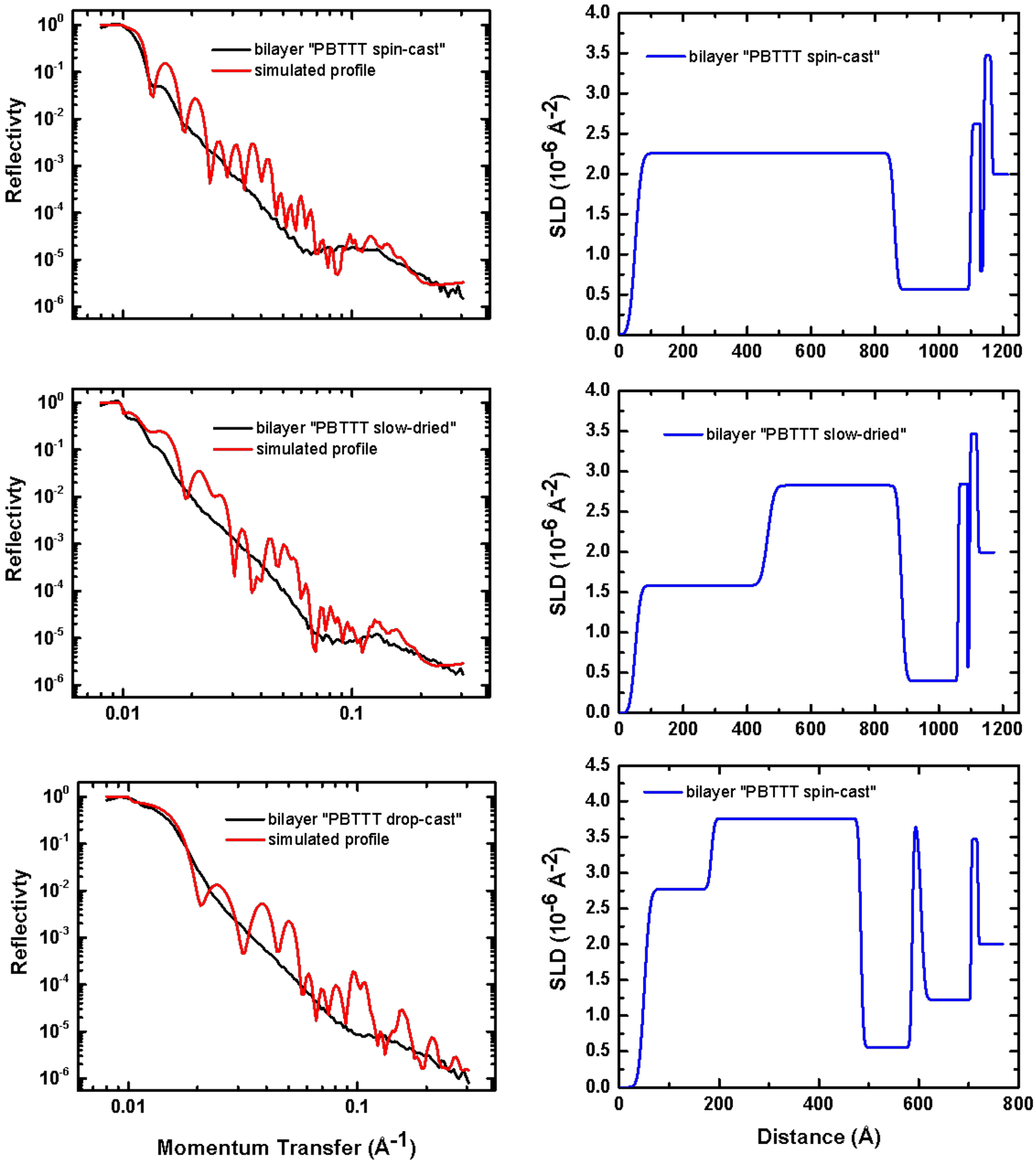

Supplementary figure S2: Simulated NR profiles for "PBTTT spin-cast", "PBTTT slow-dried" and "PBTTT drop-cast" bilayers, in which the interfacial layer roughness values are ten times lower that the experimental ones.
